# 326. Radiologic Findings of COVID-19 Associated Mucormycosis (CAM) from India

**DOI:** 10.1093/ofid/ofab466.528

**Published:** 2021-12-04

**Authors:** Aswath Govindaraju, Deepti H Vijayakumar, Raghavendra Tirupathi, Jaffar A Al-Tawfiq, Ali A Rabaan

**Affiliations:** 1 Sparsh Superspecialty Hospital, Bangalore, Bengaluru, Karnataka, India; 2 Columbia Asia Referral Hospital, Bangalore, Bengaluru, Karnataka, India; 3 WellSpan Health, Chambersburg, Pennsylvania; 4 Johns Hopkins school of medicine, Dhahran, Al Bahah, Saudi Arabia; 5 Johns Hopkins Aramco Health Care, Dhahran, Al Bahah, Saudi Arabia

## Abstract

**Background:**

The unique feature of the second wave of the COVID -19 pandemic in India has been the alarming surge of acute invasive fungal infection among COVID -19 patients. The increased incidence of rhino-orbito-cerebral mucormycosis is a matter of concern, as this fulminant infection has high morbidity and mortality. Hence, it is imperative to understand it’s imaging features, for early diagnosis, staging and treatment.

**Methods:**

We systematically reviewed 32 COVID-19 cases with imaging diagnosis of acute invasive fungal rhino-sinusitis or rhino-orbital-cerebral disease between March to May 2021. These patients underwent contrast MRI of the paranasal sinus, orbit and brain. Contrast enhanced CT chest and paranasal sinuses were done as needed.

**Results:**

The age group ranged between 30 to 71 yrs with male preponderance. The most common predisposing factors were intravenous steroid therapy and supplemental oxygen. All cases were confirmed by fungal culture and most common was Mucor. The rhino-orbito-cerebral mucormycosis was staged as below

In our study we found that the most common site in the nasal cavity was the middle turbinate /meatus and the earliest sign was non-enhancing / “black” turbinate. Premaxillary and retroantral fat necrosis was the earliest sign of soft tissue invasion. Spread via the sphenopalatine foramen and pterygopalatine fossa was more common than bony erosions. Orbital cellulitis and optic neuritis were the most common among stage 3 cases. Of patients with CNS involvement, the most common were cavernous sinus thrombosis and trigeminal neuritis. Two patients with pulmonary mucormycosis showed large necrotic cavitary lesions, giving the characteristic “bird’s nest” appearance.

Figure 1. Black turbinate

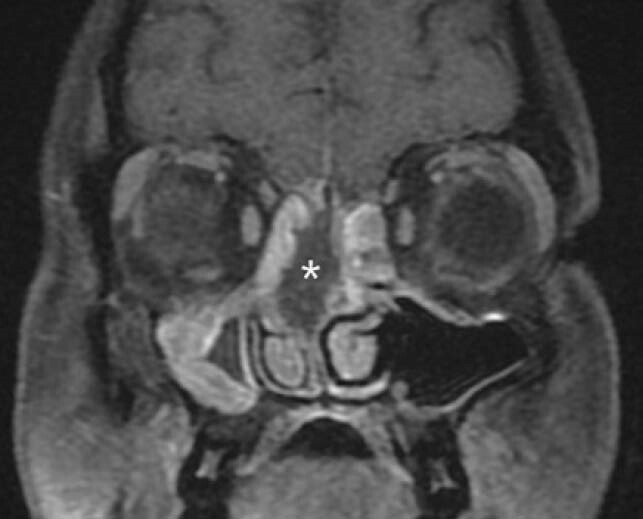

Contrast enhanced coronal T1 FS images of paranasal sinuses shows necrotic non-enhancing right superior and middle turbinates (*)

Figure 2: Axial contrast enhanced T1 FS image showing necrotic non enhancing premaxillary (arrowhead) and retroantral fat (straight arrow) walled off by thin enhancing rim.

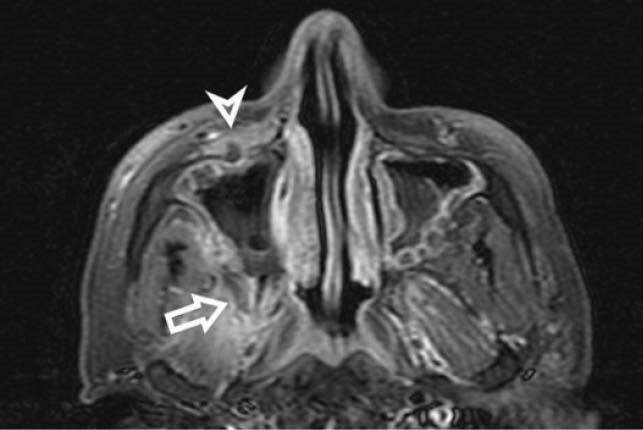

Figure 3: Contrast enhanced axial T1 FS images of paranasal sinuses shows necrotic non-enhancing left middle meatus spreading along sphenopalatine foramen in to pterygopalatine fossa (arrow head)

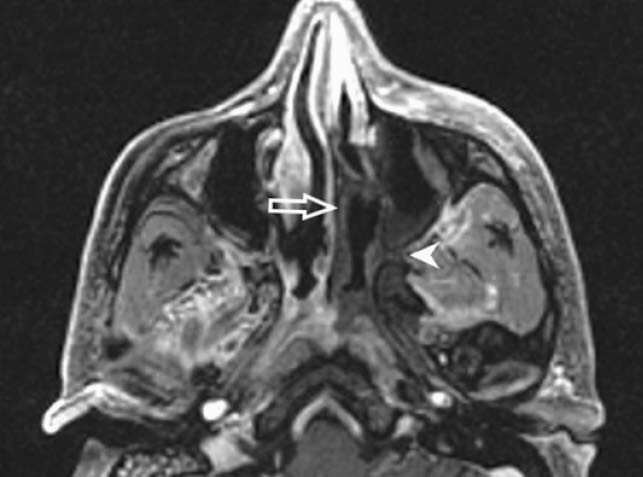

**Conclusion:**

The mortality rate was 20% in our study. In our short term follow up, 30 % of recovered patients had relapse on imaging due to incomplete clearance and partial antifungal treatment. High clinical suspicion and low imaging threshold are vital for early Mucormycosis detection in COVID-19 patients. Familiarity with early imaging signs is critical to prevent associated morbidity /mortality.

Figure 4: Contrast enhanced coronal T1 FS and diffusion weighted images shows necrotic non-enhancing left middle meatus with left orbital cellulitis (*) and optic neuritis (white arrow)

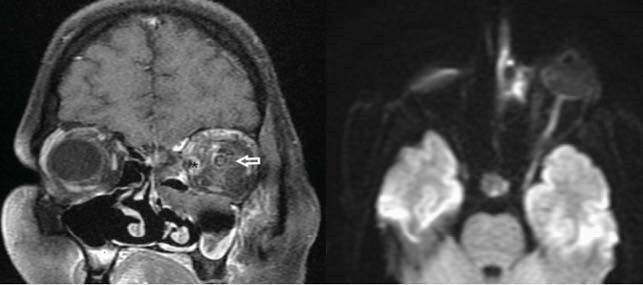

Figure 5. Bird’s nest

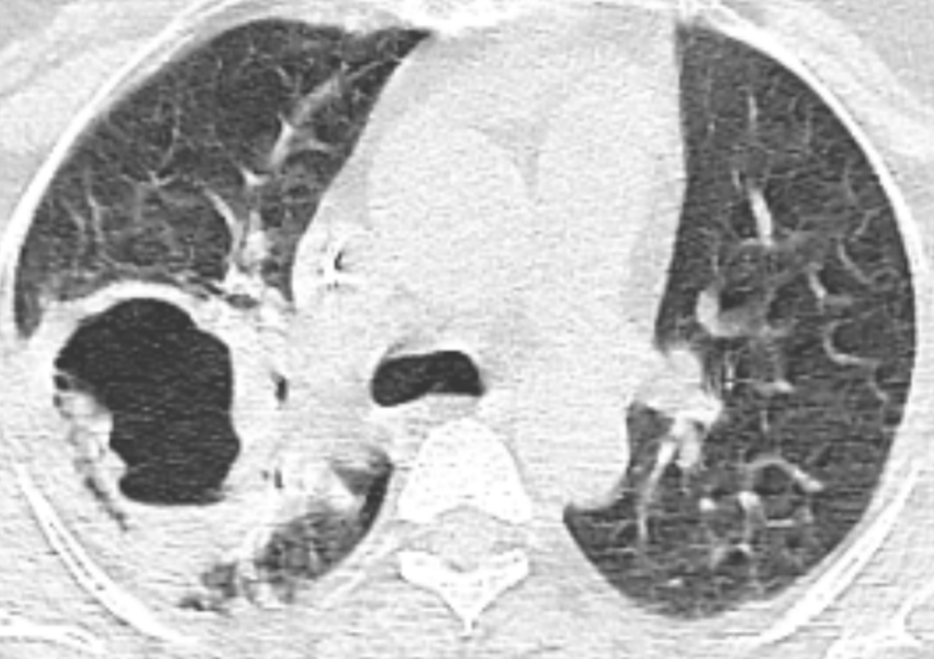

Axial CT chest image in lung window shows necrotic right upper lobe cavity with internal septations and debris on a background of surrounding COVID-19 changes.

**Disclosures:**

**All Authors**: No reported disclosures

**Table:** 

Stage	No. of cases
1 (Limited to nasal cavity)	2
2 (Involving Paranasal sinuses)	14
3 (Involvement of orbit)	8
4 (Involvement of CNS)	8

